# Alleviation of cisplatin-induced hepatotoxicity and nephrotoxicity by L-carnitine

**DOI:** 10.22038/IJBMS.2022.65427.14395

**Published:** 2022-07

**Authors:** Snur Mohammad Amen Hassan, Azad K Saeed, Omed Omer Rahim, Shler A F Mahmood

**Affiliations:** 1 Department of Anatomy and Histopathology, College of Veterinary Medicine, Sulaimani University, Sulaimani, 4601, KRG, Iraq; 2 Department of Food Science and Quality Control, Bakrajo Technical Institute, Sulaimani Polytechnic University, Sulaimani, 4601, KRG, Iraq; 3 Department of Microbiology, School of Medicine, Sulaimani University, Sulaimani, 4601, KRG, Iraq

**Keywords:** β-catenin, Cisplatin, Cyclin D, Hepatic regeneration, L-carnitine, Tubular necrosis

## Abstract

**Objective(s):**

To assess the protective effect of L-carnitine in reducing cisplatin toxicity via estimating biochemical tests, histomorphometric, and immunohistochemistry (IHC) of β-catenin and cyclin D.

**Materials and Methods:**

Fifteen adult male rabbits were used in this study and allocated into 3 groups; Group 1 (Control negative), rabbits of this group were not given any treatment. In group 2, the animals were injected with cisplatin single-dose/per week. Group 3 rabbits were treated with Cisplatin+L-carnitine orally by gavage tube for 29 days. At the end of the experiments, blood samples from all rabbits were taken from the earlobe, and then the biochemical test was done, the kidney and tissue sections were prepared for both H& E and IHC for both β-catenin and cyclin D genes.

**Results:**

Treatment with L-carnitine reduced the injury effect of cisplatin via a decline in serum creatinine, urea, bilirubin, GPT, GOP, and ALP significantly (*P<*0.05). Also, administration of LC attenuates the histopathologic abnormality in the kidney (15.71% vs 85.18%) and liver (score 3 vs 15 ) induced by cisplatin. L-carnitine elevates the expression of β-catenin and cyclin D in renal and hepatic parenchyma by diffuse, moderate-strong positivity vs cisplatin that showed local-weak staining.

**Conclusion:**

These findings imply that L-carnitine, by its pleiotropic actions in activating *Wnt* signaling, alleviates cisplatin-induced renal and hepatic destruction. It might be a method of preventing cisplatin-related nephrotoxicity and hepatotoxicity.

## Introduction

Cisplatin (cisdiammine dichloro platinum II, CDDP) is a platinum-based anti-cancer medication used for the treatment of testicular (1), cervical, ovarian, breast (2), and bladder cancers (3).

However, due to its negative side effects, its therapeutic usage is usually limited. Acute kidney damage is the most common dose-limiting side effect of cisplatin treatment (AKI). Indeed, nephrotoxicity is a common side effect of cisplatin therapy, affecting around a third of patients. However, there are no effective therapies for AKI caused by cisplatin (4). The mechanism of cisplatin-induced kidney damage is complicated by several processes that are still unknown (5). Increased generation of reactive oxygen species (ROS) and decreased expression of endogenous antioxidant enzymes have been linked to cisplatin treatment, as well as GSH reduction and lipid peroxidation, both of which cause DNA damage (6). Following cisplatin-induced AKI, tubular epithelial cells release a range of chemokines, and pro-inflammatory cells such as macrophages, and macrophages invade the damaged kidneys (7). 

L-carnitine (L-trimethyl-3-hydroxy-ammoniabutanoate) is a vitamin-like quaternary ammonium compound (8). It is composed of amino acids lysine and methionine, both of which are essential. It derives from a combination of dietary (75%) and endogenous sources (25 %) (9). L-carnitine is required for generation of ATP during oxidation of fatty acids in mitochondria (10). As a result, L-carnitine may be able to protect a variety of cell types from mitochondrial oxidative stress and death (11).

*Wnt*/β-catenin signaling enhances a variety of transcriptional signals, including cell cycle arrest, antioxidant production, and cell survival (12). *Wnt*/β-catenin signaling is expressed at low levels in the healthy adult kidney, notably in the papilla, but it is up-regulated in animal models of acute and chronic renal injury. *Wnts* and β-catenin activity were identified in the proximal tubule in the ischemia-reperfusion injury (IRI) model (13). 

Activated catenin has a slew of targets, several of which appear to be tissue-specific. Cyclin D1 is one of the most well-studied targets for activated β-catenin (14). Hepatocyte proliferation is triggered by signaling pathways that concentrate on cyclin D1, the cell cycle’s checkpoint. Cyclin D1 is an important factor in liver regeneration (15).  

The study was designed to assess the protective effect of orally administered L-carnitine against the toxic effects of cisplatin in rabbits by measuring some biochemical tests related to the liver and kidney function: serum urea, serum creatinine, bilirubin, GPT, GOT, and alkaline phosphatase levels and assessment of renal and hepatic injury by utilizing histomorphometric and IHC, focusing on the effect of β-catenin and cyclin Dfor the context of renal and hepatic regeneration and repair.

## Materials and Methods

***Animals*** ***&*** ***Housing***

Fifteen adult male rabbits (1800-2000g), aged 10-13 weeks were used in this study. This experiment was performed in the animal house of the Biology Department, School of Science, Sulaimani University, Iraq. Animals were housed in plastic cages. Five animals were kept in each cage during the experiment, and they were housed under typical laboratory settings, which included a 12:12 hr light/dark photoperiod at a temperature of 23±2 ^°^C. The animals had unrestricted access to water and food. The study was approved by the Local Ethical Committee for Animal Experimentation, College of Veterinary Medicine, University of Sulaimnai (permission 030511, 2-March-2022) based on CONCEA (National Animal Experiment Control Council) ethical norms for animal experimentation.

***Experimental design***

The animals were assigned to three groups, each with five rabbits. Group 1 (Control negative), rabbits of this group were not subjected to any treatment. Group 2 (Control positive), the animals were injected intraperitoneal (IP) with cisplatin (2 mg/kg BW), single-dose/week (16). Cisplatin was procured from Vitane Pharmaceutical inc., USA. Group 3 (Treatment group), rabbits were treated with cisplatin (2 mg/kg BW), single-dose/week intraperitoneally then given L-carnitine (Duty Pharma Company Ltd.) orally by gavage tube (20 mg/kg/day) for 29 days (17). This study was performed for about four consecutive weeks.

***Assessment of renal and liver function***

At the end of the experiments (4 weeks), blood samples from all rabbits were taken from the earlobe, and then the serum was separated. Creatinine, urea, bilirubin, GPT, GOP, and ALP were analyzed using Auto-Analyzer (LISA 200, France) colorimetric detection kit according to the manufacturer’s protocol.

***Tissue sample collection***

The animals were euthanized using (Ketamine-Xylazine: 35 mg/kg+Xylazine 5 mg/kg IM of body weight) at recommended dose intraperitoneally. Tissue samples were taken from liver and kidney tissues. The specimens were fixed for at least 24 hr in 10% neutral buffered formalin before being dehydrated in a graded series of ethanol. The tissues were dehydrated, then cleaned in xylene, and fixed in paraffin. In the Histopathology Lab of Anwar Shexa Medical City/Sulaimani Governorate, two thin sections (4 m) were mounted on glass slides and stained with hematoxylin and eosin (H&E), and one section stained for IHC markers including β-catenin and cyclin D marker. 

***Histological analysis and immunohistochemical staining***

A descriptive-analytical study was done for estimating the severity of renal tubules and hepatocytes. The tissue sections were examined and photographed (Amscope^TM^, Japan) under a light microscope. The severity of tubular injury was determined semiquantitatively by evaluating the proportion of damaged area: 0=0%; 1=<10%; 2=11-25%; 3=26-45%; 4=46-75%; and 5=76-100% (18). The tubular injury was defined as tubular cell swelling and hydropic degeneration, tubular epithelial necrosis, cast formation, intra-tubular debris, and loss of the brush border. Under 400 magnification, entire tubular numbers per field were considered normal for scoring injured tubules. Injury score (percent) ¼ (number of injured tubules/number of complete tubules)x100 was used to compute the grading percentage in each field. At least ten sections of the cortex per slide were chosen at random. (19).

Under light microscopy at 400X magnification, a histopathologist analyzed the liver slides blindly (Olympus light microscope, Japan). Histological photomicrographs were analyzed for semi-quantitative evaluation of liver damage, and liver grading was done according to the extent of sinusoidal dilatation, inflammatory cell infiltration, congestion, degeneration, or cytoplasmic vacuolization, and proliferation of Kupffer cells, and hepatic necrosis was studied. For each rabbit, 10 fields were randomly assessed. Data were scored as 0=normal, 1=mild, 2=moderate, or 3=severe (20).

***Immunohistochemical analyses***

For immunohistochemistry evaluation, serial sections of 4 mm thickness were taken on day 30, tissues were soaked in paraffin wax. Paraffin-embedded slides were dewaxed in xylene and hydrated. Sections were heated for 20 min in a microwave oven in a 10 mM sodium citrate buffer (pH 6.0) and then cooled in de-ionized water. Endogenous peroxidase activity was reduced by incubating the slices in 3 percent hydrogen peroxide for 10 min. Primary antibodies were incubated overnight at 4 ^°^C with β-catenin and cyclin rabbit polyclonal antibody (1:600; DAKO, Denmark). The reaction was enhanced by using biotin-labeled anti-rabbit secondary antibodies and streptavidin linked to horseradish peroxidase, as directed by the manufacturer (DAKO Cytomation, USA). Diaminobenzidine was used to see the reaction products (Sigma-Aldrich Co., St. Louis, MO, USA). Hematoxylin counterstained, dehydrated as per usual method, and coated with coverslips.

The nuclei were not stained remaining blush color, while the cytoplasm was stained with brownish granules of β-catenin and cyclin D. Computer-assisted image analysis software was used to examine slices under a microscope (Motic, Japan) (Am ScopeTM Version 2.5 software, Japan). Each kidney and the liver portion were inspected under a microscope at 400X magnification.

No staining or 0 for 5% positive staining, (1) for 5-25 percent positive staining, (2) for 25-50 percent positive staining, (3) for 50-75 percent positive staining, and (4) for >75 percent positive staining were used to quantify the degree of positively stained epithelial cells in IHC staining of β-catenin and cyclin D. The intensity of β-catenin and cyclin D staining was rated on a scale of mild or weak (+1), moderate (+2), moderate-strong (+3), and strong (+4). The positive reactivity extent and level of staining intensity were multiplied to get a total staining score, which ranged from 0 to 16. 

***Statistical analysis***

For statistical analysis, differences between the groups were tested by using ANOVA in SPSS ver. 22 program. A *P*-value <*0.05* was considered significant. All data were expressed as the mean±standard deviation (SD).

## Results

***L-carnitine attenuated cisplatin-induced kidney injury (AKI)***

According to biochemical tests, the rabbits in the control negative group showed normal ranges for kidney enzymes as seen in Table 1. 

Rabbits were administered 2 mg/kg of cisplatin for inducing AKI. All rabbits survived throughout the experiment after cisplatin injection. As shown in Table 1, cisplatin treatment increased serum urea and creatinine levels significantly (*P*<0.0*5*), indicating the development of acute renal failure in the cisplatin-injected rabbit. However, administration of L-carnitine mainly reduced the elevated levels of serum urea significantly vs cisplatin (50.80±1.64 vs 74.00±3.08, *P*<0.0*5*). Additionally, treatment with L-carnitine reduced the injurious effect of cisplatin via a decline in serum creatinine (1.32±0.04 vs 1.76±0.040, *P*<0.0*5*), while versus control negative group cisplatin and L-carnitine showed an increase in the enzyme levels by severe-mild degrees, respectively as seen in Table 1.

**Figure 1:**
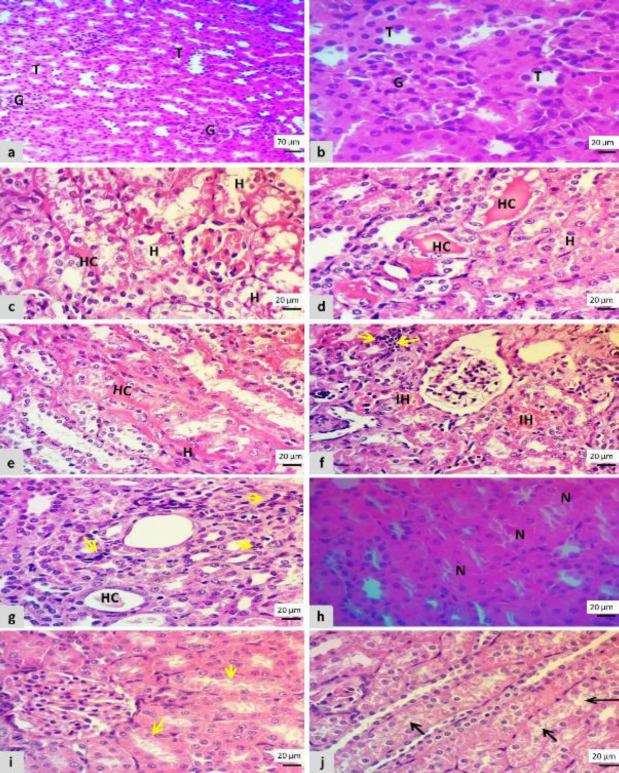
Microscopic features of kidney section showed

Histopathological features of the kidney section in the control negative group revealed normal morphology of glomeruli, proximal and distal convoluted tubules with Henley loops, and healthy renal vasculature (Figures 1A, B).

A microscopic examination of the kidney that was treated with cisplatin revealed obvious tubulointerstitial damages that included glomerular congestion and degeneration, dilation of Bowman’s space, tubular degeneration including marked swelling in the lining epithelium of renal tubules, and loss of brush border with severe vacuolation of renal tubules or ballooning degeneration, interstitial hemorrhage, tubular necrosis that showed clear pyknotic nuclei and eosinophilic cytoplasm, eosinophilic proteinaceous material (hyaline cast) in the lumen of renal tubules and Henley loops, also the lumen filled with necrotic debris accompanied by focal-interstitial infiltration of mononuclear inflammatory cells (Figures 1C-H).

**Figure 2:**
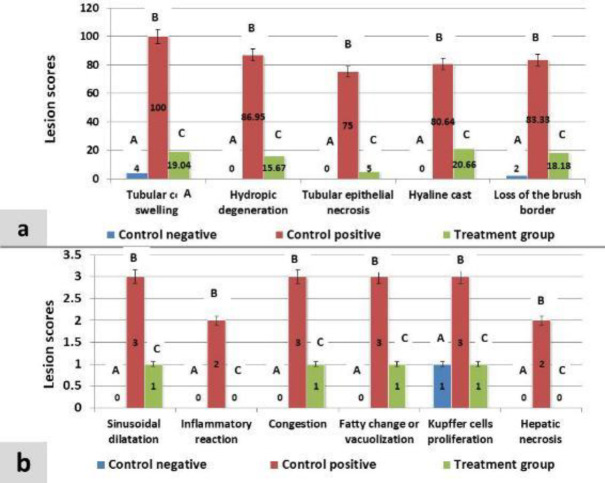
Column chart showing; A: Percentages for each kidney lesion in control negative, cisplatin, and L-carnitine groups. B: Scores for the liver parenchyma’s lesions in all groups. Scores with different alphabetical letter superscripts are significantly different (*P<*0.05)

While the kidney section that was treated with L-carnitine showed considerably decreased tubular degeneration vs cisplatin group and revealed slight swelling of glomeruli, swelling of the lining epithelium of collecting tubules that formed a star-shaped appearance, and also swelling of the epithelial lining of Henley loops in which their cytoplasm became rarefied (Figures 1I, J). Regarding the total scoring of tubular injury the L-carnitine reduced the tubular injury vs cisplatin group, (15.71% vs 85.18%), in contrast to the control negative group that showed the lowest score (1.2), both groups revealed the highest score as seen in Figures 2A.

***L-carnitine attenuated cisplatin-induced liver injury***

All liver enzyme levels were increased in the cisplatin group and LC group vs the control negative group (Table 1). L-carnitine ameliorates the bilirubin level vs the cisplatin group significantly (0.06±0.00 vs 0.09±0.00, *P*<0.05). Also, L-carnitine significantly reduced GPT vs CP group, (81.80±1.93 vs 111.60±9.88, *P*<0.05). The L-carnitine group showed significant decreases vs the cisplatin group in the GOT level (100.80±1.62 vs 114.20±1.68, *P*<0.05). Regarding ALP, a non-significant difference was recorded in both cisplatin and L-carnitine groups. In comparison with the control negative group, cisplatin and L-carnitine elevated enzyme levels by a severe-mild degree, as shown in Table 1.

**Figure 3:**
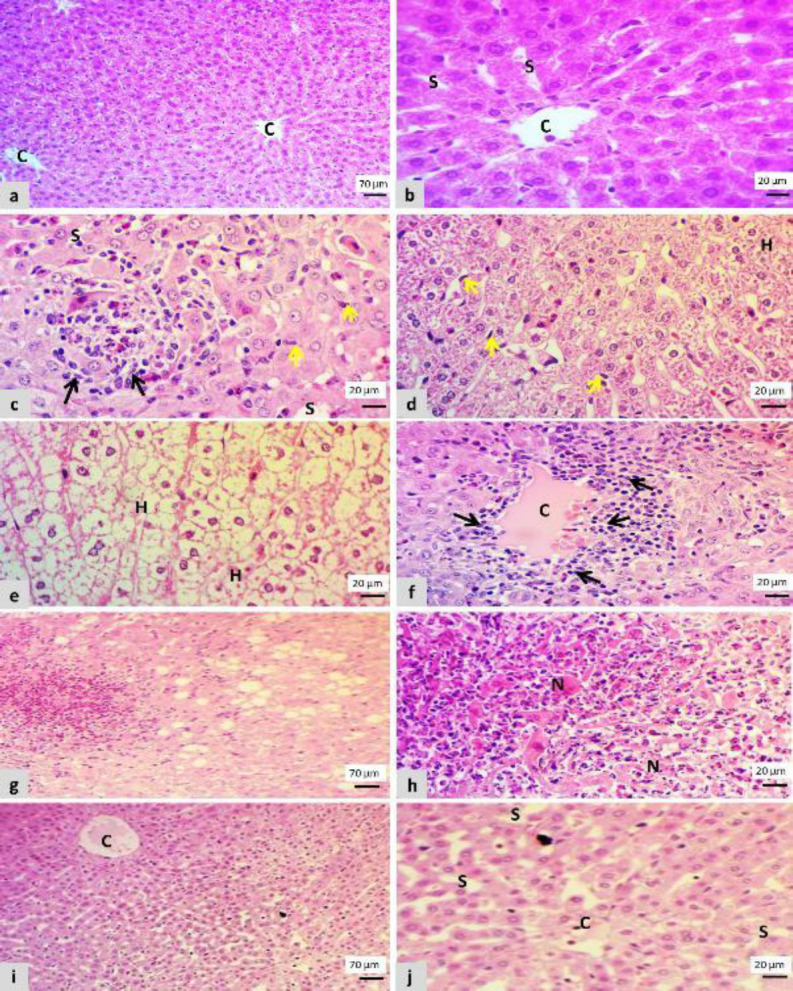
Microscopic features of a liver section showed;

The liver section in the control negative group showed normal organization of central vein and hepatocytes with intact histological features, besides normal hepatic circulation (Figures 2A, B). The histological changes of the liver in the group that was treated with cisplatin showed marked congestion of the central vein and sinusoidal dilation, hepatocellular degeneration peculiarly hydropic and fatty degeneration, presence of focal mononuclear infiltrations near the central vein, also inside the sinusoid that leads to hepatic cord disorganization with the proliferation of kupffer cells and focal infiltration of cells in periportal triad region (Figures 3A-F). 

The cisplatin-plus-L-carnitine group (Figures 3G, H) had a marked decrease in hepatocellular degenerations that only showed slight central vein congestion with sinusoidal dilation and swelling of hepatocyte vs the cisplatin group.

Concerning the total scoring of hepatic injury, the L-carnitine declined the hepatic lesions vs cisplatin group, (4 vs 16), respectively, while in comparison with the control negative group the scores in both groups were increased (Score=1 vs 4:16) in which the score for each lesion is shown in Figure 2B.

***Immunohistochemistry interpretation***

**Figure 4:**
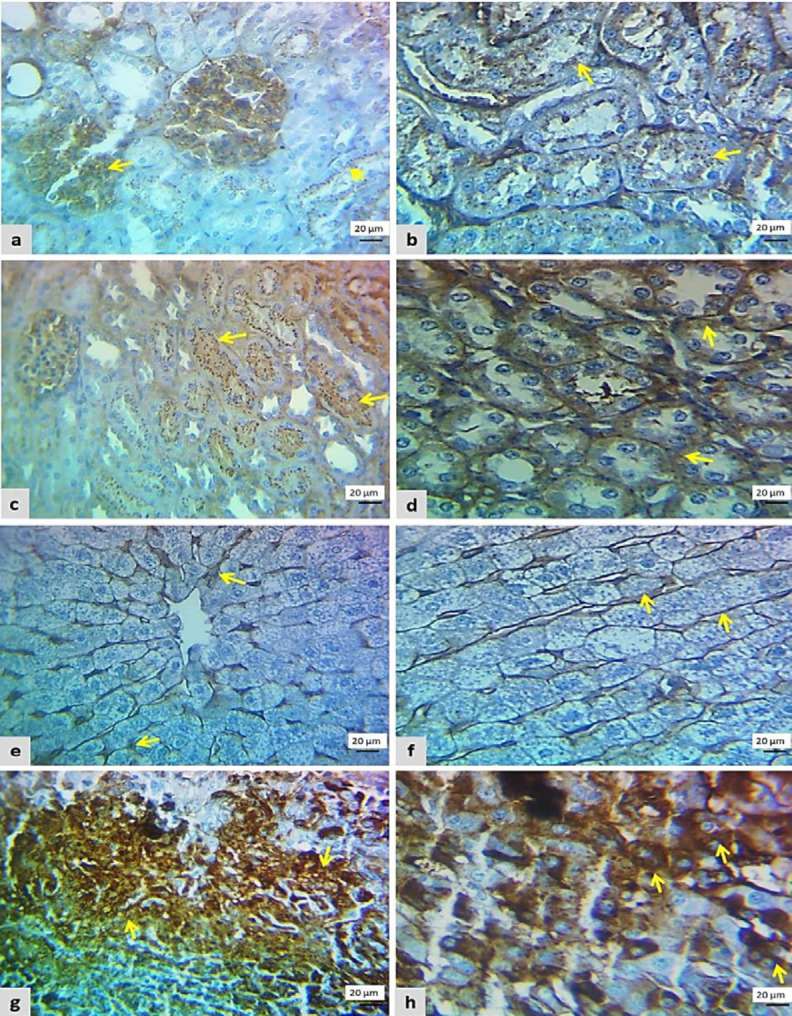
Photomicrograph of β-catenin cytoplasmic immunostaining from all examined groups

**Figure 5:**
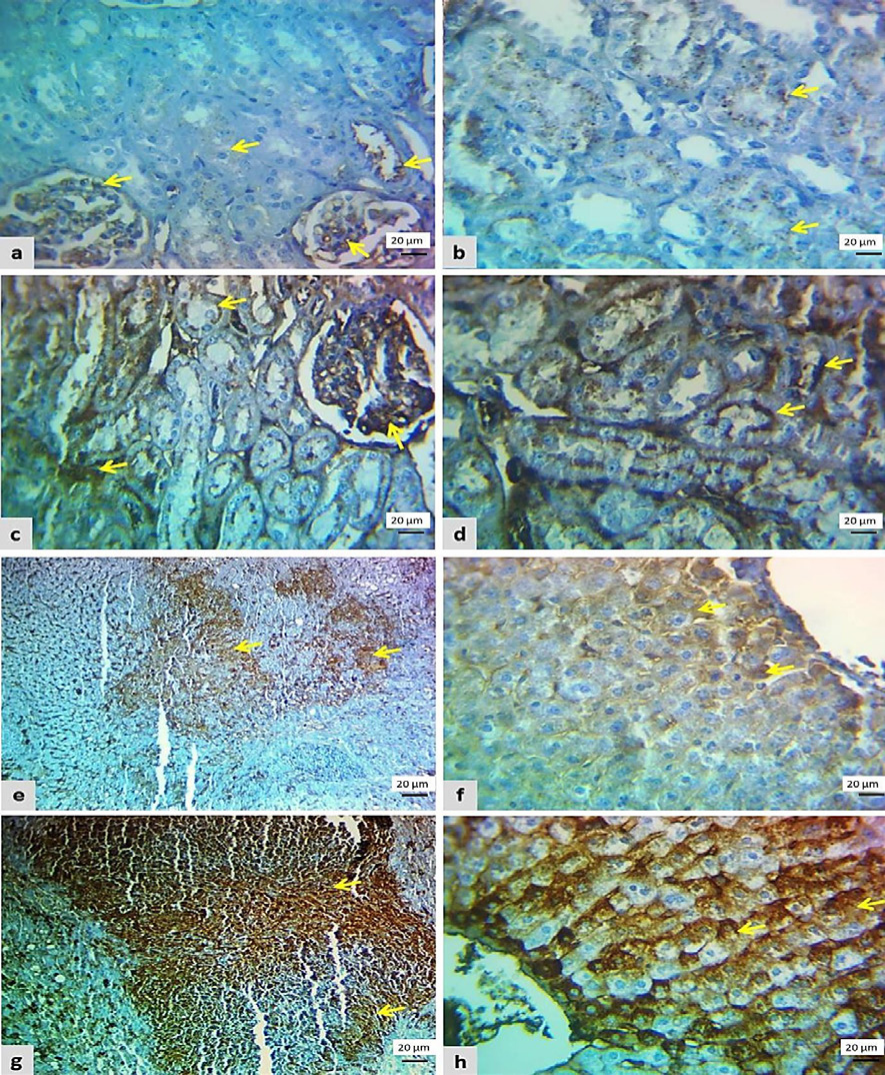
Photomicrograph of cyclin D1 cytoplasmic immunostaining from both groups. A: Focal-weak staining (score 2) in the kidney section of the cisplatin group. B: Diffuse moderate-strong staining (score 9) in the kidney section of the L-carnitine group. C: Focal-weak staining (score 2) in a liver section of the cisplatin group. D: Diffuse moderate-strong staining (score 16) in the liver section of the L-carnitine group

Immunohistochemically, no positive cells were seen in the control negative group regarding β-catenin and cyclin expression. In the cisplatin group, the renal cells showed weak brownish cytoplasm (score 2) for β-catenin and cyclin D immunolabeling in more than 25% of renal parenchyma (Figures 4 and 5A, B). Also, β-catenin and cyclin D expression was seen in 30% of liver parenchyma as weak immunopositive cells (score 2) as in Figures 4 and 5E, F. 

An increase in the number and intensity of β-catenin and cyclin D positive cells was identified in the L-carnitine group when compared with that of the cisplatin group, renal tissue showed moderate-strong β-catenin expression in more than 50% of renal cells (score 6) as seen in Figures 5 c,d. While the β-catenin positive cells in the liver section were found in 60% (score 9), (Figures 4g,h). Regarding cyclin D expression in the L-carnitine group, it was diffusely found in moderate-strong staining (score 9) in more than 50% of renal sections (Figures 4c,d), while it was diffusely expressed in more than 75% with moderate-strong intensity (score 16) in liver section (Figures 5G, H).

**Table 1:** Measurements of kidney and liver function enzymes in different study groups

Parameters	Groups		
	Control negative	Control positive (Cisplatin)	Treatment group (L-carnitine)
Serum urea (mmol/L)	45.00±0.70	74.00 ±3.08^***^	50.80±1.64^***^
Serum creatinine (μmol/L)	0.84±0.040	1.76±0.040^***^	1.32±0.04^***^
Bilirubin(mg/dL)	0.05±0.00	0.09±0.00^***^	0.06±0.00
GPT (U/L)	70.60±1.86	111.60±9.88^***^	81.80±1.93^***^
GOT(U/L)	56.00±2.84	114.20±1.68^***^	100.80±1.62^***^
Alkaline phosphatase (U/L)	49.60±2.01	67.00±2.96^***^	63.40± 2.95^***^

All values were expressed as Mean±SE. The ANOVA test was used for the statistical analysis. *** *P<*0.05

A, B: Normal histological structures of the kidney (G) glomeruli and (T) tubules in the control negative group. C-F: Dilation of Bowman’s space, marked tubular swelling, loss of brush borders, hydropic degeneration (H) with interstitial hemorrhage (IH), and presence of hyaline cast in the tubular lumen (HC) and Henley loops in cisplatin group. F and G: Focal interstitial infiltration of mononuclear cells as indicated by yellow arrows in the cisplatin group. H: Necrosis of renal tubules with marked pyknotic nuclei with the presence of eosinophilic renal tubule cytoplasm (N), in the cisplatin group. I and J: Slight swelling of glomeruli and renal tubules (yellow arrows), with ballooning degeneration of renal tubules and Henley loops as indicated by black arrows in the L-carnitine group (H& E stain)

A-B: Normal features of the liver (C) central vein and (S) sinusoidal capillary in the control negative group. C-F: Swelling of hepatocytes (S), dilation of sinusoidal capillary, proliferation of Kuffer cells (yellow arrows), severely vacuolated hepatocytes have wispy cleared and distending cytoplasm appearance that leads to loosing of hepatocytes row (H), congestion of central vein (C), focal mononuclear infiltration near the central vein and inside the sinusoidal capillary (black arrows) in cisplatin group. G: Marked fatty degeneration as indicated by the F letter. H: Hepatic necrosis characterized by eosinophilic cytoplasm and pyknotic nuclei as indicated by the N letter, in the cisplatin group. I-J: Congestion of central vein (C), slight swelling (S) of hepatocytes in which their cytoplasm becomes pale-colored with centrally located nuclei in the L-carnitine group (H& E stain)

A: Focal-weak staining (score 2) in the kidney section of the cisplatin group. B: Diffuse moderate-strong staining (score 6) in the kidney section of the L-carnitine group. C: Focal-weak staining (score 2) in a liver section of the cisplatin group. D: Diffuse moderate-strong staining (score 9) in a liver section of the L-carnitine group

## Discussion

Due to the incredible potential for treating a wide range of cancers, cisplatin’s clinical use is typically restricted due to its severe toxic side effects, which can compromise therapeutic efficacy (21). 

In the present work, cisplatin significantly increased blood urea and creatinine levels vs the control negative group, which indicates the nephrotoxic effect of cisplatin. Similar results were reported by another study (22). Nephrotoxicity is caused by the toxic effect of cisplatin on renal tubules, which inhibits specific membrane transport systems, increasing the excretion of several essential endogenous substances. This causes a sharp decline in excretory mechanisms within the kidney, increasing the accumulation of byproducts such as urea, nitrogen, and creatinine (23). In this investigation, we found that L-carnitine can ameliorate the harmful effects of cisplatin on kidney function by reducing oxidative stress and lowering creatinine and urea levels, implying that carnitine may play a role in kidney protection. This is in line with a former study, which found that L-carnitine’s capacity may be related to its antioxidant effect and ability to operate as a free radical scavenger, which protects membrane permeability (24).

The current study looked into nephrotoxicity in rats given cisplatin, which showed substantial abnormalities in the renal parenchyma, which matched a recent discovery: distinct degenerative alterations in the proximal and distal convoluted tubules, including Henley loops and tubular epithelial desquamation (22), congestion, and dilatation of interstitial blood vessels and capillaries as reported by Neamatallah *et al*. (2018) (25). Moreover, the presence of eosinophilic hyaline casts in some renal tubules is approved by another study (26). In addition, some glomeruli were found to be damaged, with a widening of glomerular spaces, in agreement with other research (27). Cellular uptake and accumulation, inflammation, oxidative stress, vascular injury, endoplasmic reticulum (ER) stress, necrosis, and apoptosis have all been proposed as reasons for its nephrotoxicity (28). According to a previous study, renal injury is frequently caused by nephrotoxic injury to kidney tissue, which results in tubular necrosis due to creation of covalent connections between a reactive metabolite of the parent drug and cell protein or DNA, culminating in tubular necrosis (29). It has also been demonstrated that cisplatin induced the inflammatory process and generation of inflammatory cytokines via activation of the NF-B, poly ADP-ribose polymerase-1 (PARP-1), and toll-like receptors (TLRs) pathways (30). In this study, we found that L-carnitine can restore functional indices and renal pathological injury in the injured kidney, allowing it to exert beneficial effects against cisplatin-induced nephrotoxicity by decreasing the score for histological changes vs the cisplatin group, which had the highest score.

The present investigation discovered several biochemical and histological changes in the liver which indicate cisplatin-induced hepatotoxicity. Hepatocellular damage was revealed by substantially increased serum enzyme activity compared with the control negative group, which included Bilirubin, GPT, GOT, and alkaline phosphatase, similar results were recorded by other research (31). The liver absorbs a large amount of cisplatin and stores it in the hepatocyte, causing damage and an increase in liver enzyme activity (32). Because they are released into the bloodstream when the hepatocyte plasma membrane is compromised, increased liver enzyme activities are recognized to be indicators of cellular leakage and loss of functional viability of hepatocytes (33). It was discovered that membrane rigidity, lipid peroxidation, cardiolipin oxidative damage, and glutathione depletion were all involved in cisplatin-induced hepatotoxicity (34). Because Carnitine decreases oxidative stress, it has a protective effect with antioxidant and anti-inflammatory characteristics, lowering bilirubin, GPT, GOT, and alkaline phosphatase levels (35). Current data showed that cisplatin caused severe morphological changes in liver tissues, including sinusoidal dilatation, congestion, and central venous dilatation, as previously stated (36). Parenchymal inflammation, hepatocyte cytoplasmic vacuolation, and proliferation of Kupffer cells were findings in the experimental studies conducted by others (37, 38).   Our findings imply that L-carnitine can protect the liver from cisplatin-induced histological alterations with the highest score via decreasing scoring degree and also greatly improving and normalizing liver histology, as seen by nearly normal hepatocytes and sinusoids. El-Shitany and Eid (2017) found similar results, demonstrating that L-carnitine can reduce the hepatotoxic effects of cisplatin (39). 

The current study’s immunohistochemistry findings are consistent with the biochemical and histopathological lesion parameters being different between the experimental groups. To the best of our knowledge, this is the first study to look into the strong protective effect of L-carnitine consumption on the renal and liver tissues by activating the proliferative and regeneration characteristics of β-catenin and cyclin D to improve damages caused by cisplatin.

In the present study, cisplatin caused a significant decrease in renal and hepatic expression of β-catenin and cyclin D that was expressed in a weak intensity localized extension (score 2), therefore a severe lesion was detected in the renal sections of rabbits that were treated with cisplatin only. The basis for our interpretation is a prior study that found that *Wnt*/ β-catenin signaling is protective in the context of kidney injury and that the favorable effects are most likely mediated by the proximal tubule. GSK-3, the kinase that targets β-catenin for degradation, has been shown to promote kidney recovery following cisplatin-induced injury in studies (40). Tubular epithelial cells are likely protected by *Wnt*/ β-catenin signaling, which modulates the production of pro/anti-apoptotic proteins to promote tubule persistence (41). Low expression of β-catenin in the cisplatin group causes changes in renal cytoarchitecture that are insufficient to restore the damage. One of β-catenin’s downstream target genes is cyclin D1, which binds to cyclin-dependent kinases (CDK) 4 and 6 to promote cell cycle progression (42). Conversely, following cisplatin-induced nephron and hepatic toxicity, treatment with L-carnitine dramatically increased the expression of β-catenin and cyclin D in renal tubules by the moderate-strong intensity and diffuse positive cells, which are proliferative proteins, and promoted regeneration from damage (40). Overexpression of β-catenin increased cyclin D1 protein expression and sped up cell cycle progression in LLC-PK1 cells, supporting our hypothesis that L-carnitine groups have less kidney injury (43). The previous study also highlighted that this signaling pathway is also required for the maintenance of a toxin-repairing unipotent renal progenitor cell (41). 

Because cyclin D1 is essential in triggering hepatocyte proliferation and restoring liver mass following partial hepatectomy, our results suggested that L-carnitine has a hepatoprotective role via expression of cyclin D1. Cyclin D1 maintains its normal pathway in cell cycle progression in hepatocytes (44). Cyclin D1 expression correlated with DNA synthesis, indicating that cyclin D1 plays a function in hepatocyte cell cycle progression (45).

## Conclusion

Our research is the first to demonstrate that L-carnitine may have a role in cisplatin-induced nephrotoxicity and hepatotoxicity by activating the *wnt*/β-catenin pathway. Our theoretical and experimental findings suggest that L-carnitine may control renal and hepatic injury by speeding up the expression of β-catenin and cyclin D, two proteins that provide regeneration and repair processes, hence reducing functional histological changes.

## Authors’ Contributions

SMA, AKS, and AAF conceived and designed the experiment; SMA and AAF performed the experiments; SMA and OOR wrote the manuscript.

## Conflicts of Interest

The authors declare no conflicts of interest.
